# Revision shoulder arthroplasty and proximal humeral bone loss: a comprehensive review and proposal of a new algorithm of management

**DOI:** 10.1186/s10195-024-00784-0

**Published:** 2024-09-03

**Authors:** Angelo Baldari, Luca Saccone, Antonio Caldaria, Edoardo Giovannetti de Sanctis, Gian Mauro De Angelis D’Ossat, Luca La Verde, Alessio Palumbo, Francesco Franceschi

**Affiliations:** 1 Department of Orthopaedic and Trauma Surgery, San Pietro Fatebenefratelli Hospital, Rome, Italy; 2 Faculty of Medicine and Surgery, Link Campus University, 00165 Rome, Italy; 3 Department of Orthopaedics and Traumatology, Fondazione Policlinico Universitario Campus Bio-Medico of Rome, 00128 Rome, Italy; 4 Institut Universitaire Locomoteur et du Sport (IULS), Hôpital Pasteur 2, CHU de Nice, 30, avenue Voie Romaine, 06000 Nice, France

**Keywords:** Classification system, Proximal humeral bone loss, Revision shoulder arthroplasty

## Abstract

With the rising prevalence of shoulder arthroplasty, the incidence of revision shoulder arthroplasty is also increasing. The complexity of these revision procedures poses significant challenges, with bone loss being a critical factor impacting treatment outcomes. Addressing substantial humeral bone defects is crucial for ensuring implant stability and functionality. A comprehensive literature review was conducted using PubMed, Medline, and Google Scholar to identify existing classification systems for proximal humeral bone loss in the context of revision shoulder arthroplasty. The study assessed the advantages and limitations of these classifications, using this information to propose a new diagnostic and therapeutic algorithm. Several classification systems for proximal humeral bone loss were identified. McLendon et al. classify proximal humeral bone loss based on a 5-cm bone loss threshold and suggest an allograft prosthesis composite for losses exceeding this limit. Boileau’s system stratifies bone loss into four types based on the extent of loss, with specific recommendations for each category. The PHAROS classification provides a detailed anatomical assessment but lacks quantitative precision. The proposed PHBL-SCORe system offers a novel algorithm incorporating preoperative radiographic measurements to determine the percentage of bone loss and guide treatment options. Proximal humeral bone loss presents significant challenges in revision shoulder arthroplasty, necessitating precise preoperative planning and classification to guide surgical intervention. Existing classification systems provide valuable frameworks but often rely on average population values, neglecting individual anatomical variations. The proposed PHBL-SCORe system offers a patient-specific approach, improving the accuracy of bone loss assessment and optimizing treatment strategies. Implementing this classification in clinical practice could enhance surgical outcomes and reduce complications associated with rRSA (revision Reverse Shoulder arthroplasty). Further studies are required to validate this algorithm and explore its long-term efficacy in diverse patient populations.

## Introduction

With the increasing prevalence of shoulder arthroplasty, the occurrence of revision total shoulder arthroplasty (rTSA) is on the rise [1]. Initially recommended for patients with cuff tear arthropathy, reverse total shoulder arthroplasty (RSA) indications now encompass various elective degenerative diagnoses. These include a massive irreparable rotator cuff tear with or without glenohumeral osteoarthritis, rheumatoid arthritis, chronic dislocations, postinfectious sequelae, tumor resection, and the revision of failed anatomical or hemiarthroplasties. rTSA has consistently posed challenges, yielding only limited satisfactory results historically [2]. The complexity of revision procedures often presents a challenge for surgeons in identifying the key factors that influence the treatment [3], among which the issue of the bone loss is usually the prevalent one. Surgeons often find themselves compelled to address a significant defect in the humeral bone in instances involving not only the revision of a failed shoulder arthroplasty but also a complex fracture, periprosthetic fractures, nonunions, periprosthetic infection, and humeral resection for tumors. It is known that the proximal humerus ranks as the third most common site for osteosarcoma and the second most common site for all osseous sarcomas, with the majority necessitating surgical intervention [4]. Limb salvage and reconstructive surgical procedures have become prevalent treatments for these cases [5].

For all these reasons, the issue of proximal humeral bone loss (PHBL) is crucial, and it emphasizes the importance of the surgeon’s ability to anticipate such defects for proper fixation and stability [4]. PHBL compromises both proximal bony fixation and the potential for ingrowth into the humeral stem. As a result, the implant relies on rotational stability within the diaphysis. This situation has been shown to increase rotational micromotion, suggesting a heightened risk of stem loosening, dislocation, weakness, and—potentially—worse function [5]. There is significant variability in the severity of bone loss encountered during surgery; in these scenarios, employing a classification system for PHBL can significantly support surgeons by guiding diagnosis, treatment, and prognosis.

Several authors have investigated PHBL in the context of rRSA (revision Reverse Shoulder arthroplasty). Nevertheless, there remains no consensus on a thorough and reliable approach. This study seeks to offer an outline of existing classification systems for PHBL linked to rTSA and to suggest a novel algorithm for managing such surgical cases.

## Materials and methods

A comprehensive search encompassing PubMed, Medline, and Google Scholar was conducted, utilizing various combinations of keywords such as ‘humeral classification’ along with ‘bone loss,’ ‘defect,’ ‘revision,’ and ‘shoulder arthroplasty.’ All peer-reviewed journals were surveyed, and articles detailing classification systems for PHBL associated with rTSA were analyzed. Additional cross-referencing of the selected articles was conducted to identify further relevant literature for the study. Furthermore, the authors proposed a new classification, which underwent evaluation for inter- and intra-observer reliability utilizing weighted kappa coefficients as assessed by two independent orthopedic surgeons. The level of agreement was interpreted according to the Landis and Koch [6] criteria, where a score exceeding 0.80 denotes excellent agreement, 0.61–0.80 indicates good agreement, 0.41–0.60 signifies moderate agreement, 0.21–0.40 suggests fair agreement, and 0.20 or lower indicates poor agreement.

### The McLendon et al. classification

**Fig. 1:**
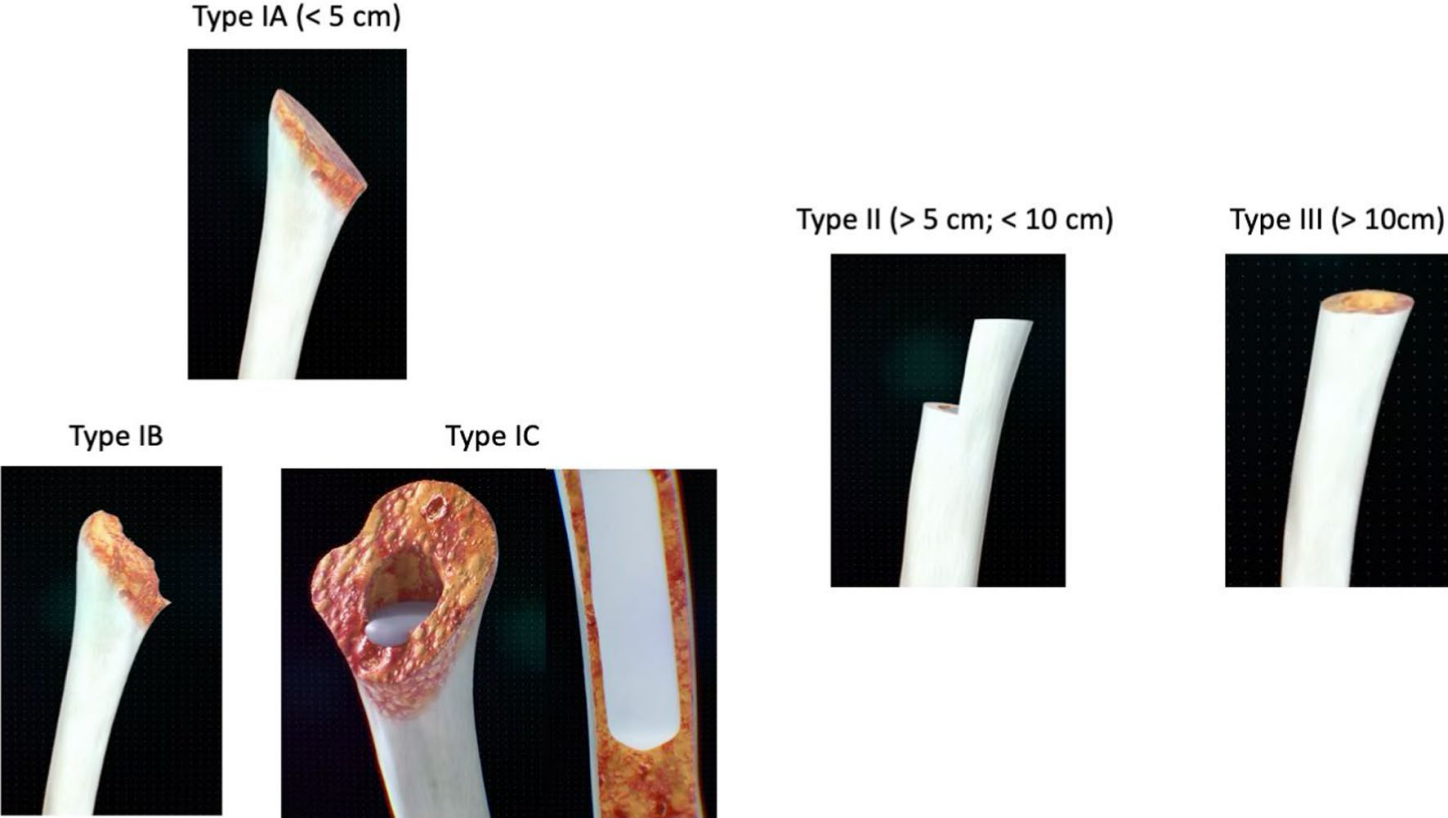
This classification is an upgrade of McLenon et al. classification

In 2017, McLendon et al. [7] introduced surgical indications based on whether bone loss was measured as being less than or exceeded 5 cm from the top of the medial humeral tray to the medial humeral shaft. The authors suggest considering an allograft prosthesis composite (APC) [8] once this 5-cm threshold is surpassed. This indication, initially developed to predict the need for a larger diaphyseal allograft affecting the deltoid insertion, was later expanded by Cox and McLendon [9] to cover all bone loss types in revision surgery (Fig. 1). It categorizes PHBL into several types: type I, less than 5 cm of bone loss, typically no allograft needed; type IB, asymmetrical loss, less than 5 cm medially, more laterally; type IC, intact cement mantle that needs revision for rotational stability; type II, 5–10 cm of loss, shorter allografts recommended; type III, over 10 cm of loss with a compromised deltoid. Humeral stem diameter and the cement-within-cement technique were also assessed for failure rates.

### Boileau’s classification

**Fig. 2:**
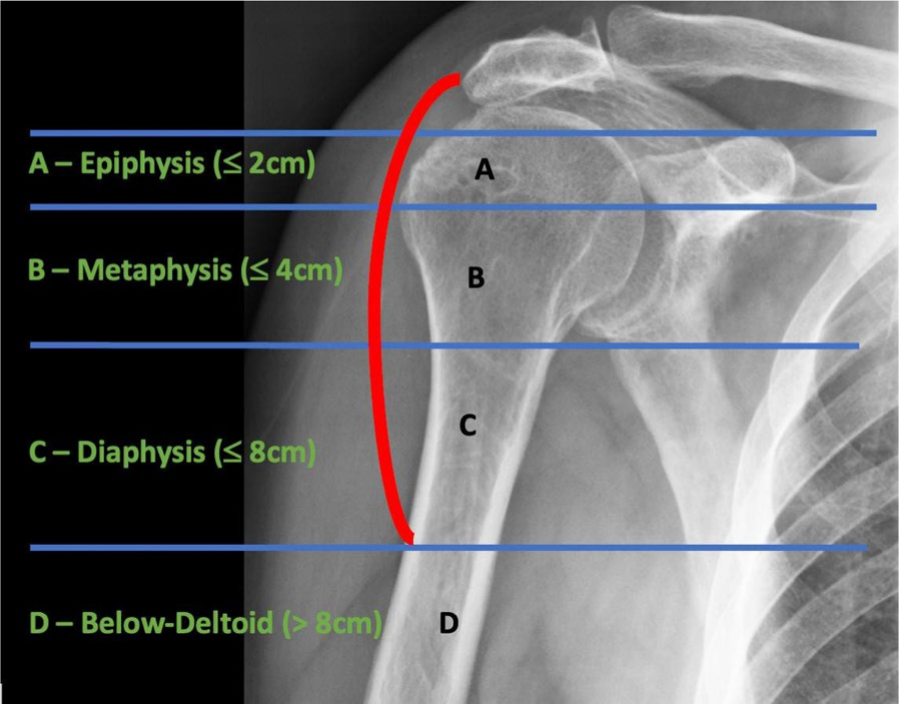
Schematic representation of the Boileau classification.

In 2018, Boileau [10] suggested a classification system based on three types of PHBL: type A, with < 2 cm of epiphyseal bone loss; type B, with < 4 cm of metaphyseal bone loss; and type C, with > 4 cm of bone loss extending into the diaphysis. Introducing this stratification, he recommended using a 4-cm threshold to distinguish between cementoplasty reconstruction and APC, while minor humeral shortening can be addressed by adjusting the glenosphere, liner, or humeral tray. In 2020, Boileau [11] provided a modification of the previously described classification, basically changing type C to > 4 cm of bone loss extending into the diaphysis but < 8 cm of bone loss (above the “V” deltoid insertion) and adding type D, which corresponds to > 8 cm of bone deficit (below the V deltoid insertion). This modification was necessary as all cases analyzed exhibited more than 4 cm of PHBL, necessitating the subdivision of the cohort into two distinct subgroups (Fig. 2). This classification system has long served as a valuable reference for shoulder surgeons preparing for rTSA. However, these indications rely on specific absolute values that may not universally apply to all patients due to anatomical size variations.

### The PHAROS classification

In 2018, a consensus group introduced the PHAROS classification [12]. This classification emerged from a comparative retrospective radiographic case series evaluation, aiming to anticipate the complexity of humeral reconstruction.

The objectives of this study were to establish a classification system for PHBL in rTSA based on plain radiographs alone and to provide therapeutic information to the surgeon. In the context of rTSA, this classification system can assist surgeons in making crucial clinical decisions, including the choice of using allograft, a higher liner, and/or higher polyethylene, cementoplasty, or massive tumoral prosthesis.

**Fig. 3:**
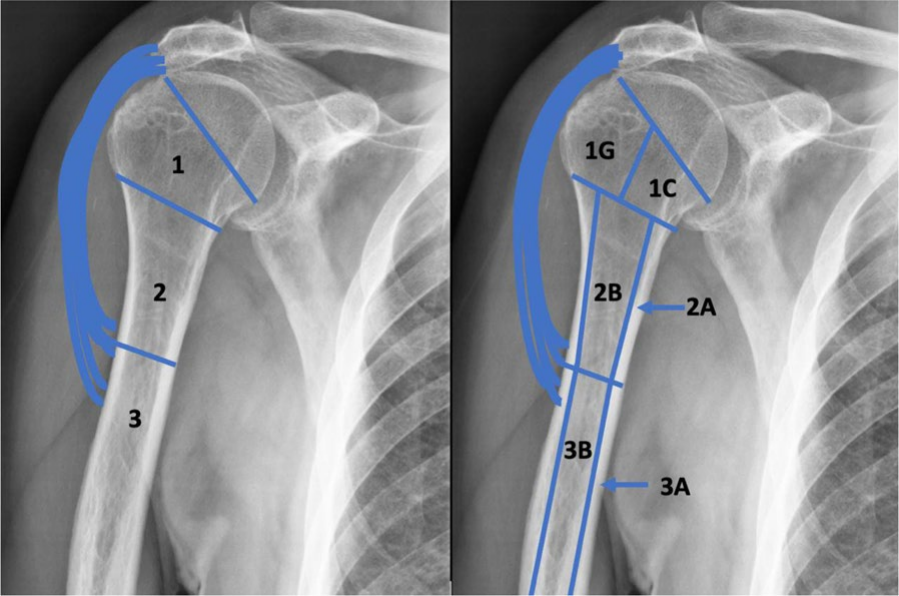
Schematic representation of the PHAROS classification

PHAROS type 1 describes epiphyseal bone loss encompassing the articular surface, tuberosities, and calcar. The classification further distinguishes between type 1C, indicating calcar loss, and type 1G, indicating loss or malunion of the greater tuberosity. Type 1 constitutes approximately 31% of all rTSA cases. PHAROS type 2 describes metadiaphyseal bone loss situated proximal to the deltoid attachment. Subtype 2A denotes cortical thinning of the metadiaphysis exceeding 50% of the expected cortical thickness, while subtype 2B involves the loss of both metadiaphyseal bone proximal to the deltoid and epiphyseal bone. PHAROS type 3 describes diaphyseal bone loss extending below the deltoid attachment. Subtype 3A indicates cortical thinning of the diaphysis exceeding 50% of the expected cortical thickness, while subtype 3B means that most of the diaphysis is compromised and there is epiphyseal and metadiaphyseal bone loss (Fig. 3).

The PHAROS classification system acts as an algorithm that provides a framework for surgical planning. For type 1C and type 1G defects, stem fixation through cement augmentation alone may be a valid option, leaving the stem proportionately elevated to restore height. Special attention is required for type 1G defects to restore anatomical tuberosity positioning and ensure excellent fixation. Type 2A defects may necessitate the consideration of a proximal humeral allograft, depending on the quality and nature of the native bone encountered intraoperatively. More definitive plans should be made for type 2B defects, likely involving the use of a proximal humeral allograft. Type 3A defects might require a long proximal humeral allograft or a femoral allograft, which can extend through the metadiaphysis. Type 3B bone defects are ideally treated with massive tumoral prosthesis.

### The PHBL-SCORe

The Proximal Humeral Bone Loss—Specific Classification for Optimal Reconstruction (PHBL-SCORe) is a novel diagnostic and therapeutic algorithm proposed by the authors. This classification system was designed to categorize proximal humeral bone loss in a clear, easily reproducible, and tailored method, with the aim of suggesting the most suitable treatment approach for each patient.

For the preoperative planning protocol, patients underwent an AP radiograph of the entire humerus, including both the limb scheduled for surgery and the unaffected limb. Radiographs were uniformly obtained at the same center, and all the radiographs were conducted following the same standardized radiographic protocol described by Lädermann et al. [13].

Radiographic measurements were obtained from scaled bilateral preoperative true antero-posterior images of the humerus. Those images were taken with patients standing and their shoulders in neutral rotation. Careful alignment of the humeri against the cassette ensured optimal image capture, with the X-ray beam focused on the middle third of the humerus. To account for magnification, a marker of known diameter was placed adjacent to the lateral humerus on the skin. Three primary lines were marked on the films for measurement in the healthy humerus and served as the “reference unit:” the trans-epicondylar line (line* y*), the diaphyseal axis (axis* t*), and a line that was perpendicular to the diaphyseal axis and passed through the highest point of the greater tuberosity. The segment of axis *t* between these perpendiculars was measured as *m*.

Three other three primary lines were marked on the film for measurement in the affected humerus: the trans-epicondylar line (*y*2), the diaphyseal axis (axis *t*2), and a line that was perpendicular to the diaphyseal axis and passed through the most prominent point of the residual proximal cortex. The segment of axis* t*2 between these perpendiculars was measured as *m*2.

**Fig. 4:**
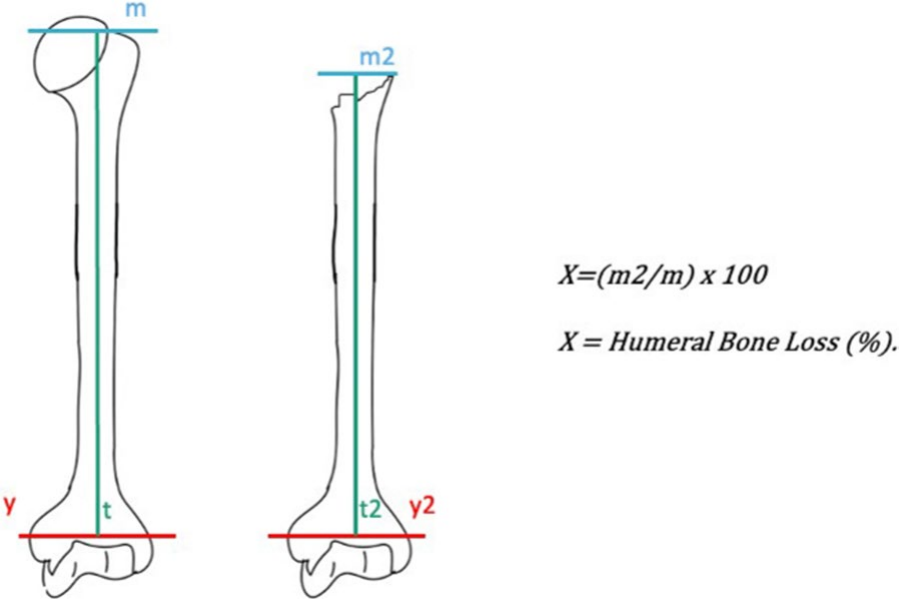
Schematic representation of the measurement of proximal humeral bone loss expressed as a percentage

**Fig. 5:**
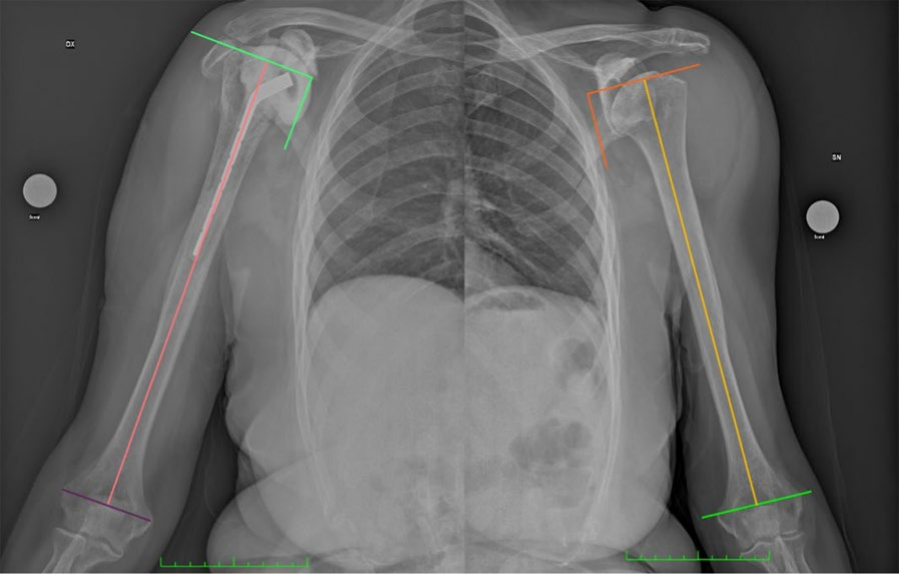
True anteroposterior bilateral scaled radiographs of humeri, captured with neutral rotation and the patient in a standing position. The humeri were laid flat against the cassette, and the X-ray beam was precisely directed at the middle third. To account for magnification, a centimeter marker was strategically positioned laterally to the humerus

The recorded values of *m* and *m*2 were used to extrapolate the predicted percentage of bone loss using the following formula: %bone loss = (*m*2/*m*) × 100 (Figs. 4, 5).

Two authors independently examined all the radiographs in a blinded manner using the Horos^®^ viewer. Measurements were individually collected by each author following the established protocols. Subsequently, the gathered data were grouped together and compared to assess their reproducibility.

The inter-reliability evaluation between the two orthopedic surgeons expressed as the kappa coefficient was 0.798.

Based on this preoperative algorithm, our recommendation for classification and surgical treatment is the following:

Type A: Proximal humeral bone loss < 5%; treatment: increase in size of the metal liner and/or polyethylene
Type B: Proximal humeral bone loss > 5% and < 15%; treatment: increase in size of the metal liner and polyethylene + cementoplasty
Type C: Proximal humeral bone loss > 15% and < 40%; treatment: APC/minimal tumoral prosthesis
Type D: Proximal humeral bone loss > 40%; treatment: massive tumoral prosthesis.

### Reconstruction options

#### Revision reverse shoulder arthroplasty without allograft

**Fig. 6:**
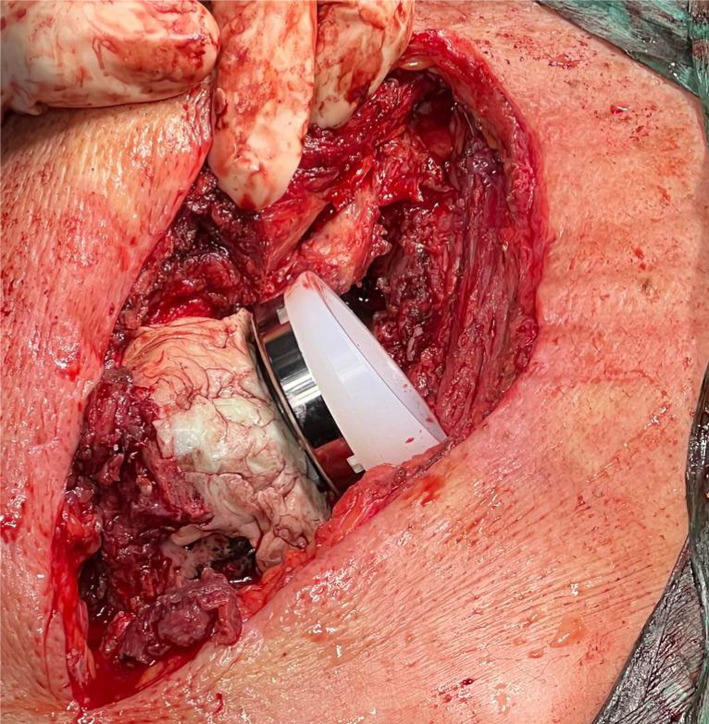
Cementoplasty reconstruction

In cases where bone defects are confined to the epiphysis, opting for a cemented revision without employing an allograft is a viable approach. The adequacy of the length can be assessed intraoperatively by using a trial component before cementation, considering factors such as soft-tissue and axillary-nerve tension, the force needed for inferior subluxation of the humerus, and the force required for prosthetic dislocation. Various methods can be employed to restore humeral length. One straightforward technique involves cementing the humeral stem in a proud position determined during preoperative planning and trialing. However, depending on the implant system, there are alternative approaches to modify the functional length of the humerus, such as metal augmentation at the stem–bone interface or utilizing a thicker polyethylene tray. Additionally, adjusting the size of the glenosphere or using an inferiorly eccentric glenosphere can influence joint stability and soft-tissue tension, compensating for minor deficits in humeral length. In some instances, a cementoplasty reconstruction may be necessary, involving the creation of a cement collar around the implant to restore the correct wrapping of the deltoid muscle (Fig. 6).

#### Allograft prosthesis composite using a proximal humeral allograft

**Fig. 7:**
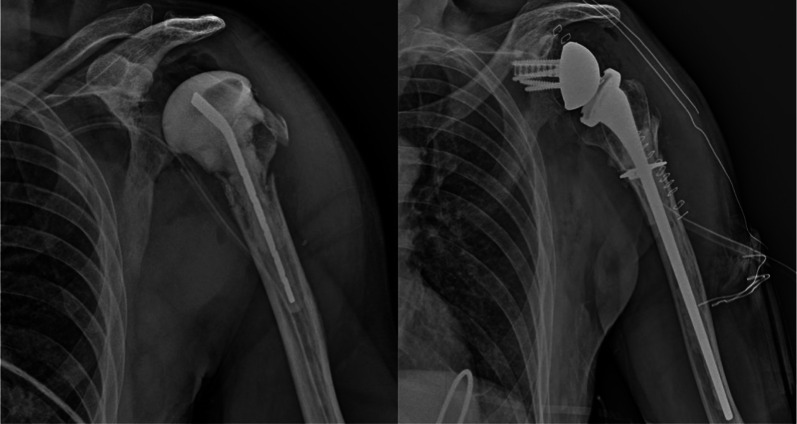
Preoperative radiograph with an antibiotic-loaded spacer and postoperative radiograph of a revision shoulder prosthesis with allograft reconstruction

In cases where bone defects extend into the meta-diaphysis, employing a proximal humeral allograft proves to be an excellent method to address bone loss and provide structural reinforcement to the humeral stem. The advantages of utilizing a proximal humeral allograft include enhanced structural support for the component, restoration of the humeral length and lateral offset, regeneration of bone stock, and attachment sites for soft-tissue structures such as the posterior cuff, subscapularis, and deltoid. Moreover, in the context of rTSA, the increased lateral offset offered by the allograft may positively impact the wrapping of the deltoid muscle. Following the removal of the previous humeral stem and a more accurate assessment of bone loss using trial implants, the preparation of the proximal humeral allograft can commence. This involves making a cut at the anatomic neck of the allograft, just distal to the articular surface, which can be achieved using either a freehand technique or a cutting guide. Subsequently, the humeral canal is reamed and broached, ensuring the preservation of sufficient cancellous bone for cement fixation. Throughout this process, it is crucial to retain the tendon stumps on the allograft for a future repair to native tendons. An estimation of the required length of the allograft for reconstruction should be made preoperatively using radiographs with magnification markers. However, final adjustments regarding stem height and the size of the allograft should be made intraoperatively based on the total bone loss and soft-tissue tensioning. Once the necessary length of the allograft has been determined, the distal humeral cut is made, which may include a step cut to enhance torsional stability. To achieve compression at the graft–host junction, a 3.5-mm locking compression plate may be utilized in compression mode across the junction, ensuring the selection of a plate with sufficient holes proximal and distal to the junction. Otherwise, a cerclage wire at the site of the step cut may be employed to provide additional fixation (Fig. 7). Any preserved native tendons (such as the posterior cuff, deltoid, and pectoralis) should then be repaired to their respective allograft tendon stumps.

#### Revision reverse shoulder arthroplasty with a massive tumoral prosthesis

While primarily employed in the oncologic setting for the wide resection of proximal humerus malignancies, a massive tumoral prosthesis can also be effectively utilized to address extensive bone defects. Surgeons often prefer this type of implant over osteoarticular allografts or allograft–prosthesis composites due to the latter's increased risk of nonunion and graft failure [14]. The highly modular design of these prostheses offers a relatively straightforward approach to restoring humeral length, and achieving an appropriate version is typically feasible through the use of the modular components. Native tendons can subsequently be reattached to the prosthesis using suture fixation, although the efficacy of soft-tissue healing to metal remains uncertain.

## Discussion

rTSA often poses significant technical challenges and frequently yields outcomes that do not match those observed in primary arthroplasty [1]. The primary reasons for rTSA, listed in descending order, include instability, infection, humeral loosening, and glenoid loosening [15]. A common cause of instability is postoperative humeral shortening [15]. This complex and high-risk surgical procedure is associated with complications and reoperation rates ranging from 10 to 50% [3]. Several studies [4, 7, 10, 12, 16, 17] have explored the issue of PHBL in the context of revision shoulder arthroplasties. Proximal humeral bone loss can compromise proximal bony fixation of the humeral stem during rTSA; consequently, the implant depends on rotational stability within the diaphysis. This condition has been demonstrated to elevate rotational micromotion, indicating an increased risk of stem loosening [5].

We analyzed various studies [4, 7, 9, 10, 12, 16] that address this issue and suggest surgical indications and classification systems; however, these often rely on average values derived from the general population, disregarding individual patient variations such as height, sex, and other distinguishing characteristics. For instance, the previously established 4-cm cutoff for humeral bone loss, used by Boileau [9] to determine the need for an APC, might be excessively restrictive for taller individuals. This quantitative cutoff lacks individual specificity and thus fails to account for unique patient characteristics. The existing literature lacks high-quality randomized prospective trials, and there is a lack of consensus regarding the optimal reconstructive technique following PHBL.

Chacon et al. [8] initially stated that the choice to employ a proximal humeral allograft was not predetermined before the surgery; rather, the decision to utilize an allograft during the operation was determined based on the extent of proximal bone loss observed coupled with an evaluation of the soft-tissue tension in the shoulder. The humeral allograft serves to enhance humeral stem stability, safeguard against humeral loosening, and restore the proximal humeral bone stock. This restoration is beneficial for maintaining the height of the prosthesis–bone construct, ensuring deltoid tension as well as providing a residual tendon stump.

While we fully approve of the considerations of the group, the tackling of a complex case such as an rTSA with PHBL should not excessively rely on intraoperative assessments alone. Numerous challenges surround the decision to use an allograft, including bureaucratic procedures and waiting times for graft requests, the increase in the costs of the procedure, the need for timely defrosting that may compromise its use for another patient, the time required for appropriate bacteriological checks, and the availability of suitable operating room facilities. Considering these challenges, it becomes evident that understanding how a surgical procedure should be performed is crucial before commencement. This necessitates precise guidelines that are adaptable to the variability of each patient, accounting for differences in the amount of bone deficit and the high anatomical variability among patients.

Studies from the scientific literature [18–20] revealed significant variability in humeral length among individuals. The authors discovered that the mean maximum humeral length was 304.56 ± 14.16 mm in males and 276.60 ± 10.89 mm in females. Hertel et al. [20] analyzed 200 humeri and noted lengths ranging from 24.5 to 36.8 cm, with an average length of 31.6 ± 2.3 cm.

For example, analyzing the data gathered by Hertel et al. [20], we observed that the 5 cm of bone loss required for an APC when planning an rTSA corresponds to 20.4% of the bone loss for a small humerus, 15.8% for a medium humerus, and 13.5% for a large humerus. These findings can be extrapolated to other surgical considerations involving more or less bone loss.

These findings indicate that the cutoff employed by McLendon [7, 9] or Boileau [6] to assess humeral bone loss might exhibit too much variability for individuals with extremely long or short humeri. Such variability in assessment could lead to inaccurate evaluations of bone loss, potentially resulting in inappropriate surgical decision-making and subsequent reconstruction failure.

The significance of these variations becomes even more pronounced when considering the dynamic perspective of implant stability influenced by muscle insertions, which may be compromised or absent in specific bone loss scenarios. Moatshe et al. [19] emphasized that the deltoid’s footprint is, on average, 70.1 mm from the humeral apex, with a range of 64.6–75.7 mm. The extension area averages 732 mm^2^, with a range of 621 mm^2^ to 843 mm^2^. Similar variations are observed in the insertions of other tendons crucial to the shoulder kinematics, such as the pectoralis major or latissimus dorsi. In meticulous and individualized surgical planning, it is essential to acknowledge the presence of similar variations in human anatomy.

The PHAROS classification system [12] shows adaptability to individual patients by assessing proximal humeral bone loss based on compromised anatomical zones. Although qualitative values are used to identify the defect area, specificity in quantifying the extent of compromise is lacking. As a result, significant disagreements among the reviewers were observed. There was a 35% (38 out of 108) discrepancy among the reviewers regarding alphanumeric grades and a 29% (31 out of 108) disparity concerning numeric grades in shoulders. Moreover, the reviewers disagreed on multiple occasions, with a 29% (31 out of 108) mismatch in alphanumeric grades and a 23% (25 out of 108) discrepancy in numeric grades. This level of interpersonal discordance is considered not ideal. For clear guidance in treating specific clinical scenarios, meticulous and comprehensive outlining is essential.

In our view, the essential characteristic necessary for this purpose is the adoption of a straightforward and easily reproducible numerical measurement. These principles guide our proposed method for classifying and treating proximal humeral bone loss. Our goal was to create a personalized classification system for proximal humeral bone loss, offering tailored treatment approaches. This classification system proves particularly beneficial for patients situated at the extremes of the Gaussian curve, outside statistical normality.

## Conclusions

PHBL is a commonly faced issue during rTSA [21]. Given the intricate nature of this procedure, meticulous preoperative planning is imperative to mitigate even minor errors that could lead to substantial consequences. Proper evaluation of PHBL by revision surgeons is crucial to ensure appropriate fixation and stability and, ultimately, satisfactory results. A comprehensive classification system is vital for understanding the clinical nuances of each patient, identifying potential pitfalls, and providing guidance to minimize complications and failures. The proposed classification system is crafted to classify PHBL in a straightforward, highly reproducible, and patient-specific manner, thereby recommending the most appropriate treatment approach. Implementing this classification system in clinical practice could assist surgeons in managing the diverse range of humeral bone loss issues encountered in rTSA.

## Data Availability

Not applicable to a narrative review.

## Abbreviations

rTSA: Revision total shoulder arthroplasty
RSA: Reverse total shoulder arthroplasty
PHBL: Proximal humeral bone loss
APC: Allograft prosthesis composite
PHBL-SCORe: Proximal Humeral Bone Loss—Specific Classification for Optimal Reconstruction
